# Real-world data of HER2-negative early breast cancer patients treated with anthracycline and/or taxane regimens in Japan

**DOI:** 10.1007/s12282-024-01572-8

**Published:** 2024-04-29

**Authors:** Akihiko Shimomura, Yasuaki Sagara, Ryo Koto, Masakazu Fujiwara, Yuka Kanemura, Hiroshi Kitagawa, Shigehira Saji

**Affiliations:** 1 Department of Breast and Medical Oncology, National Center for Global Health and Medicine, Tokyo, Japan; 2 Department of Breast and Thyroid Surgical Oncology, Social Medical Corporation Hakuaikai Sagara Hospital, Kagoshima, Japan; 3 Medical Department, AstraZeneca K.K., Osaka, Japan; 4 Department of Medical Oncology, Fukushima Medical University, 1 Hikariga-oka, Fukushima, Fukushima 960-1295 Japan

**Keywords:** Anthracycline, Early-stage breast cancer, Luminal-type, Taxane, Triple-negative

## Abstract

**Background:**

Anthracycline- and taxane-based chemotherapy regimens are established treatments for human epidermal growth factor receptor (HER)2-negative early-stage breast cancer with high risk of recurrence. This study examined the prevalence of these chemotherapy regimens as perioperative therapy, the patterns of retreatment, and factors influencing prescription choices in Japan.

**Methods:**

This observational cohort study focused on high-risk early-stage breast cancer patients not undergoing anti-HER2 therapy, utilizing data from a hospital-based claims database in Japan spanning from April 2008 to September 2021.

**Results:**

Of 42,636 high-risk patients who underwent breast cancer surgery, 32,133 (75.4%) were categorized as having luminal-type (received endocrine therapy) and 10,503 (24.6%) as having triple-negative cancer (not receiving any endocrine therapies). Most patients (98.7%) with luminal-type breast cancer received perioperative therapy, and 40.3% of those received anthracycline/taxane. In the triple-negative group, 57.0% of all patients received perioperative therapy and of those, 93.4% received anthracycline/taxane. Being over 40 years old, having an early stage (clinical stage ≤ II), and receiving treatment in non-specialized facilities were associated with less use of anthracycline/taxane in the luminal-type group. For the triple-negative group, associated factors with less use of anthracycline/taxane included being over 60 years old, treatment in small hospital (capacity < 200 beds), and treatment in non-specialized facilities.

**Conclusions:**

Approximately half the patients in both the luminal-type and triple-negative groups were prescribed anthracycline and/or taxane for perioperative chemotherapy. The choice was associated with patient age, cancer stage, and the scale and specialization of the treatment facilities. This study sheds light on the current state of breast cancer treatment practices in Japan.

**Supplementary Information:**

The online version contains supplementary material available at 10.1007/s12282-024-01572-8.

## Introduction

Breast cancer is one of the most common malignancies in the world and the leading cause of cancer-related death among women [1, 2]. Compared with other malignancies in Japanese women, breast cancer has the highest age-adjusted mortality rate, and the annual trend is increasing [3].

According to a study by the National Cancer Center Japan, the number of newly diagnosed Japanese breast cancer patients in 2018 was approximately 100,000 [2, 4]. Most breast cancers are diagnosed at an early stage without distant metastases. As early-stage breast cancer treatment aims to eradicate micrometastases and provide a cure, anti-tumor drug therapy is an important addition to local surgery and radiotherapy [4].

The risk of recurrence is determined by tumor size, number of lymph node metastases, histological malignancy, Ki67 proliferation status, hormone receptor (HR) and human epidermal growth factor receptor (HER)2 expression level, vascular invasion, and mutations in cancer-related genes [5–7]. In HR-positive (+) HER2-negative (−) breast cancer, prognosis remains poor [8], with high rates (17.2%) of 5-year recurrence or death in patients bearing lymph node metastasis [9]. Triple-negative breast cancer (TNBC), defined as having < 1% expression of HRs by immunohistochemistry and no HER2 overexpression or amplification, also has a poor prognosis with a risk of recurrence within 3 years of approximately 30% [10–12]. For TNBC, therapeutic molecular targets are lacking and chemotherapy drugs have limited effectiveness.

Recently, the progress of drug treatment for HER2− breast cancer has been remarkable. The addition of therapies targeting cyclin-dependent kinase (CDK) 4/6 in HR+ breast cancer and immune checkpoint inhibitors in TNBC have been shown to be effective and are now widely used in clinical practice [13–15]. Furthermore, new molecularly targeted drugs, poly-(adenosine diphosphate-ribose) polymerase inhibitors, have shown potential as postoperative therapy to suppress breast cancer recurrence in cases where germline *BRCA1/2* genes are mutated [16]. Meanwhile, perioperative anti-tumor drugs, especially anthracycline and taxane-based chemotherapy regimens, have been established to treat HER2− early-stage breast cancer at high risk of recurrence [17–23]. The Japanese Breast Cancer Society 2022 Clinical Practice Guidelines recommend a sequential anthracycline-taxane regimen when the risk of recurrence is considered high, such as in cases with lymph node metastasis [24]. Additionally, the 2023 St. Gallen International Consensus Guidelines [25] for the treatment of early breast cancer recommends chemotherapy, including anthracycline, taxane, or both as pre- and/or postoperative therapy for TNBC, even for patients with T1c, that is, a tumor > 1 cm and ≤ 2 cm, in maximum diameter [25]. For HR+ HER2− breast cancer, perioperative chemotherapy is also recommended for patients with a higher risk of recurrence than in T1c and N0 tumors, regardless of their menopausal status [16]. However, the Japanese Breast Cancer Society 2022 Clinical Practice Guidelines recommend considering the risk of adverse events, along with the recurrence risk reduction effect, when deciding on perioperative chemotherapy regimens [24]. Currently, little is known about patients who receive anthracycline/taxane therapy in clinical practice. To address this knowledge gap, we analyzed data from a Japanese hospital-based claims database to clarify the treatment patterns and characteristics of high-risk early-stage breast cancer patients not receiving anti-HER2 therapy but undergoing perioperative systemic anthracycline/taxane therapy.

## Materials and methods

### Data source

We obtained data from a Japanese hospital-based claims database curated and maintained by Medical Data Vision Co., Ltd. (Tokyo, Japan). Diagnoses of diseases were coded according to the International Classification of Diseases, 10th revision (ICD-10). Data from > 25 million individual patient records from 473 hospitals across Japan were collected between 1 April 2008 and 30 September 2021 (the study period) [26].

### Study design

**Fig. 1:**
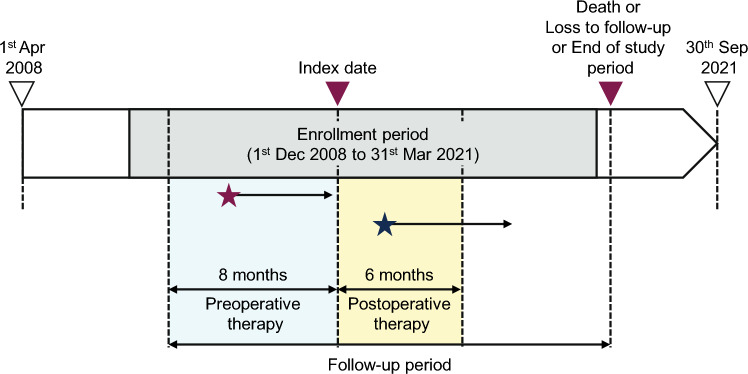
Study schema. The index date was the first date of recorded breast cancer surgery between 1 December 2008 and 31 March 2021 (the enrollment period), and the follow-up period was until death, loss of follow-up, or study end (30 September 2021), whichever came first. Treatment data were collected within 8 months before the index date for preoperative therapy, and within 6 months after the index date for postoperative therapy

This was an observational, cohort study on early-stage breast cancer patients not receiving anti-HER2 therapy who had undergone surgery for primary breast cancer. The index date was the first date of recorded breast cancer surgery between 1 December 2008 and 31 March 2021 (the enrollment period), and the follow-up period was from the index date until death, loss of follow-up, or study end (30 September 2021), whichever came first. Treatment data collection periods were 8 months before the index date for preoperative therapy and 6 months after the index date for postoperative therapy (Fig. 1). The observation period were from 8 months before the index date to the end of the follow-up period for an individual patient. Eligible patients were identified during the enrollment period.

The MINS Ethics Committees approved the study protocol (approval number: 220209). The study was conducted according to the Declaration of Helsinki and the Ethical Guidelines for Life Science and Medical and Health Research Involving Human Subjects [27]. The need for informed consent was waived.

### Patient selection

Patients were included if they had a confirmed diagnosis of breast cancer (ICD-10 code: C50.x) during the study period and a record of surgery, defined as a receipt code for primary breast cancer during enrollment in this database. The codes used for defining breast cancer diagnosis, and surgery for primary breast cancer are listed in Online Resources 1 and 2.

**Fig. 2:**
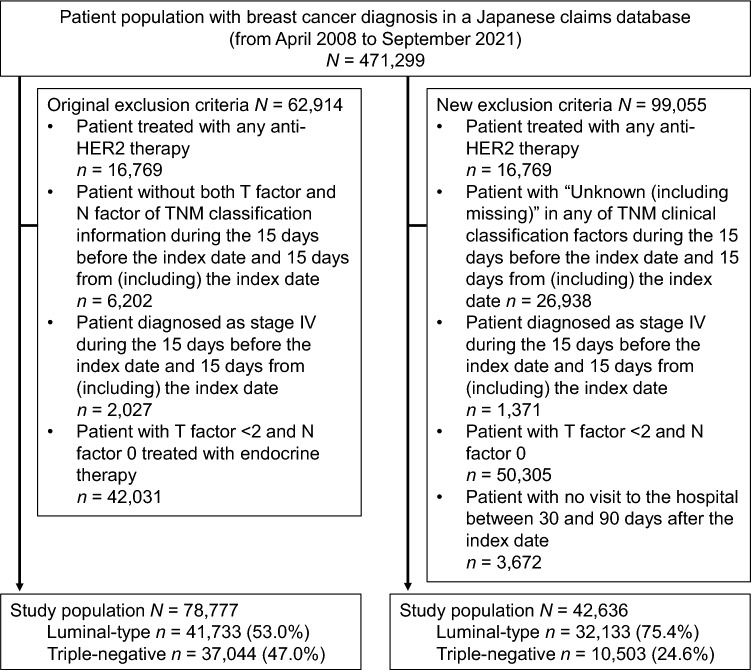
Patient disposition per original and new exclusion criteria. The original eligibility criteria yielded an unexpected study population without ‘high-risk’ disease and/or those who had insufficient follow-up (indicated in left box). Therefore, we subsequently conducted post hoc analyses using a revised set of exclusion criteria to further exclude patients (indicated in right box)

With the purpose to select only HER2− breast cancer patients at high risk of recurrence who had received perioperative treatment and whose clinical stage was known, we excluded patients who met any of the following criteria: (1) patients treated with any anti-HER2 therapy, (2) patients whose tumor (T) and node (N) factors of the TNM classification during the 15 days before the index date and 15 days from (including) the index date were unknown, (3) patients with T factor < 2 and N factor 0 treated with endocrine therapy, and (4) patients diagnosed with stage IV breast cancer based on TNM classification during the 15 days before the index date and 15 days from (including) the index date (Fig. 2). The codes used for defining drug therapy, including anti-HER2 therapy, are listed in Online Resource 3.

During the primary analyses, these eligibility criteria yielded an unexpected study population that included, for example, patients with unknown T or N factors or with T0-1 and N0, who were classified as not having ‘high-risk’ disease and never received endocrine therapy. The study population also included patients without sufficient follow-up. Therefore, we subsequently conducted post hoc analyses on a different study subset generated by a new set of exclusion criteria to further exclude patients without ‘high-risk’ disease and/or those who had insufficient follow-up. We excluded patients if they met any of the following criteria: (1) patients treated with anti-HER2 therapy, (2) patients with any ‘unknown (including missing)’ factor for the TNM clinical classification during the 15 days before the index date and 15 days from (including) the index date, (3) patients with T factor < 2 and N factor 0, (4) diagnosed with stage IV breast cancer based on TNM classification during the 15 days before the index date and 15 days from (including) the index date, and (5) patients with no hospital record from 30 to 90 days after the index date (Fig. 2).

The study population was divided into two groups, referred to as “luminal-type” or “triple-negative”, based on the medications they were receiving in the observation period rather than on pathological molecular data, which were unavailable from the data source. The luminal-type group included patients who had been treated with any endocrine therapy but no anti-HER2 therapy. The triple-negative group included patients not treated with endocrine therapy or anti-HER2 therapy.

### Covariates

Demographic data were obtained as of the month of the index date. Covariates were age, sex, TNM clinical classification, clinical stage derived using TNM classification, hospital capacity, cancer treatment center status, and the follow-up period, which were potentially associated with treatment selection for early-stage breast cancer.

### Outcomes

The primary outcome was the prescription pattern of anthracycline and/or taxane chemotherapy treatment regimens. The initial treatment pattern of preoperative therapy (initial treatment prescribed within 8 months before the index date) and postoperative therapy (initial treatment prescribed within 6 months after the index date) was categorized as a chemotherapy regimen with anthracycline, taxane, or both (including subcategories of regimens using anthracycline only, taxane only, anthracycline + taxane concurrently, and anthracycline + taxane sequentially); regimens without anthracycline or taxane chemotherapy; regimens with endocrine therapy; and regimens without chemotherapy and endocrine therapy in the triple-negative and luminal-type groups.

Secondary outcomes included the factors associated with the prescription of anthracycline and/or taxane in clinical practice and the evaluation of the treatment duration for postoperative therapy after the start of the prescription in the luminal-type and triple-negative groups. For the evaluation of the treatment duration for postoperative therapy, the number of patients censored on 30 September 2021, lost to follow-up of treatment regimen duration or death, and with prescriptions up to 24 weeks (whichever occurred first) were calculated for treatment regimen durations of 0– < 2, 2– < 6, 6– < 10, 10– < 14, 14– < 18, 18– < 24, and 24–28 weeks. Treatment regimen duration was calculated as the time from the first prescription date of initial adjuvant therapy to the date of death, the date of loss to follow-up of treatment regimen duration, the censoring date of 30 September 2021, or the date of the prescription after 24 weeks, whichever occurred first. The same outcomes were analyzed in the population during the post hoc analysis.

### Statistical analysis

As this observational study was not intended to be comparative, a sample size calculation was not conducted. Continuous variables were summarized as medians, minimums (min), and maximums (max), while numbers, proportions and 95% confidence intervals (CIs) were used to summarize categorical variables. For all statistical analyses, no missing data were imputed in this study.

Odds ratios (ORs) and 95% CIs, calculated using a multivariate logistic regression model, were estimated for prescribing chemotherapy regimens containing anthracycline, taxane, or both for the luminal-type and triple-negative groups by preoperative therapy only, postoperative therapy only, preoperative + postoperative therapy, and pre- or postoperative therapy. SAS Viya^®^ 3.5 (SAS Institute, Cary, NC, USA) was used for the statistical analyses.

## Results

### Patient demographics and baseline characteristics

During the study period, data for 471,299 patients diagnosed with breast cancer were extracted. Of these, 78,777 were eligible for the primary analyses, with 41,733 (53.0%) in the luminal-type group and 37,044 (47.0%) in the triple-negative group (Fig. 2).

The proportions of patients with unknown data, including missing data, were 25.0% in T factor, 5.8% in N factor, 6.3% in M factor, and 26.7% in the clinical stage groups (data not shown). Of the 37,044 patients in the triple-negative group, 27,278 (73.6%) patients had no pre- or postoperative therapy records, implying insufficient follow-up in this population (Online Resource 4).

The post hoc analyses using a revised study population generated using new eligibility criteria evaluated a total population of 42,636 surgical patients diagnosed with breast cancer. Of these, 32,133 patients (75.4%) were in the luminal-type group, and 10,503 (24.6%) were in the triple-negative group (Fig. 2). All the following results are from the post hoc analyses.

Table 1 summarizes the baseline characteristics of patients from the post hoc analysis population. In the overall luminal-type group (*n* = 32,133), patients had a median (min–max) age of 63 (19–100) years, and 26,679 (83.0%) and 5454 (17.0%) patients had clinical stage II and III breast cancer, respectively (Online Resource 5).

**Table 1:** Patient characteristics by new exclusion criteria

	Overall	Luminal	Triple-negative
Number of patients (*N*)	42,636	32,133	10,503
Age, years			
Median	64	63	65
Min	19	19	23
Max	100	100	100
< 40	2029 (4.8)	1409 (4.4)	620 (5.9)
40–49	8078 (18.9)	6469 (20.1)	1609 (15.3)
50–59	7546 (17.7)	5672 (17.7)	1874 (17.8)
60–69	10,095 (23.7)	7680 (23.9)	2415 (23.0)
≥ 70	14,888 (34.9)	10,903 (33.9)	3985 (37.9)
Sex			
Male	307 (0.7)	287 (0.9)	20 (0.2)
Female	42,329 (99.3)	31,846 (99.1)	10,483 (99.8)
T factor			
0	38 (0.1)	24 (0.1)	14 (0.1)
1	4967 (11.6)	4051 (12.6)	916 (8.7)
2	30,999 (72.7)	23,266 (72.4)	7733 (73.6)
3	2868 (6.7)	1972 (6.1)	896 (8.5)
4	3764 (8.8)	2820 (8.8)	944 (9.0)
N factor			
0	23,674 (55.5)	17,866 (55.6)	5808 (55.3)
1	15,368 (36.0)	11,823 (36.8)	3545 (33.8)
2	2141 (5.0)	1520 (4.7)	621 (5.9)
3	1453 (3.4)	924 (2.9)	529 (5.0)
M factor			
0	42,636 (100.0)	32,133 (100.0)	10,503 (100.0)
1	0	0	0
Clinical stage			
I	0	0	0
II	35,042 (82.2)	26,679 (83.0)	8363 (79.6)
III	7594 (17.8)	5454 (17.0)	2140 (20.4)
Hospital capacity, beds			
< 200	1371 (3.2)	1024 (3.2)	347 (3.3)
200–499	22,550 (52.9)	17,031 (53.0)	5519 (52.5)
≥ 500	18,715 (43.9)	14,078 (43.8)	4637 (44.1)
Cancer therapeutic facility status			
Yes	34,241 (80.3)	25,747 (80.1)	8494 (80.9)
No	8395 (19.7)	6386 (19.9)	2009 (19.1)
Follow-up period, weeks			
Median	157.43	168.29	120.00
Min	4.3	4.3	4.3
Max	659.4	659.4	617.9

Data are *n* (%) unless otherwise stated

*M* metastasis, *Max* maximum, *Min* minimum, *N* lymph node, *T* tumor size

In the luminal-type group, 13,526 (98.7%) patients received perioperative therapy, of whom 12,773 (94.4%) were treated with regimens including anthracyclines and/or taxane, in addition to endocrine therapy. In the triple-negative group, 5987 (57.0%) patients received perioperative chemotherapy, and of those, 5594 (93.4%) were treated with regimens including anthracyclines and/or taxane.

Patients who received chemotherapy, including anthracycline, taxane, or both, tended to be younger at a median (min–max) age of 54 (19–87) years than those who did not receive chemotherapy. Patients who underwent concurrent and sequential anthracycline + taxane regimens had median (min–max) ages of 53 (31–81) years and 53 (21–86) years, respectively. Patients undergoing only endocrine therapy had a median (min–max) age of 69 (23–100) years. Among patients undergoing chemotherapy including regimens with anthracycline, taxane, or both, anthracycline + taxane concurrent and sequential regimens, 44 (27.2%) and 2342 (31.5%) patients had clinical stage III disease, respectively. Among patients not receiving regimens with anthracycline or taxane 165 (21.9%) had clinical stage III disease.

In the overall triple-negative group (*n* = 10,503), the median (min–max) age of patients was 65 (23–100) years; 8363 (79.6%) had clinical stage II disease, while 2140 (20.4%) had clinical stage III disease (Table 1). Among patients receiving chemotherapy containing anthracycline, taxane, or both, anthracycline + taxane concurrent and sequential chemotherapy regimens had median (min–max) ages of 56 (32–85) and 57 (23–85) years, respectively (Online Resource 6). Among patients receiving concurrent (*n* = 70) or sequential (*n* = 3691) chemotherapy regimens with both anthracycline and taxane, 26 (37.1%) and 892 (24.2%) had clinical stage III disease, respectively (Online Resource 6).

### Selection of preoperative and/or postoperative therapy

**Fig. 3:**
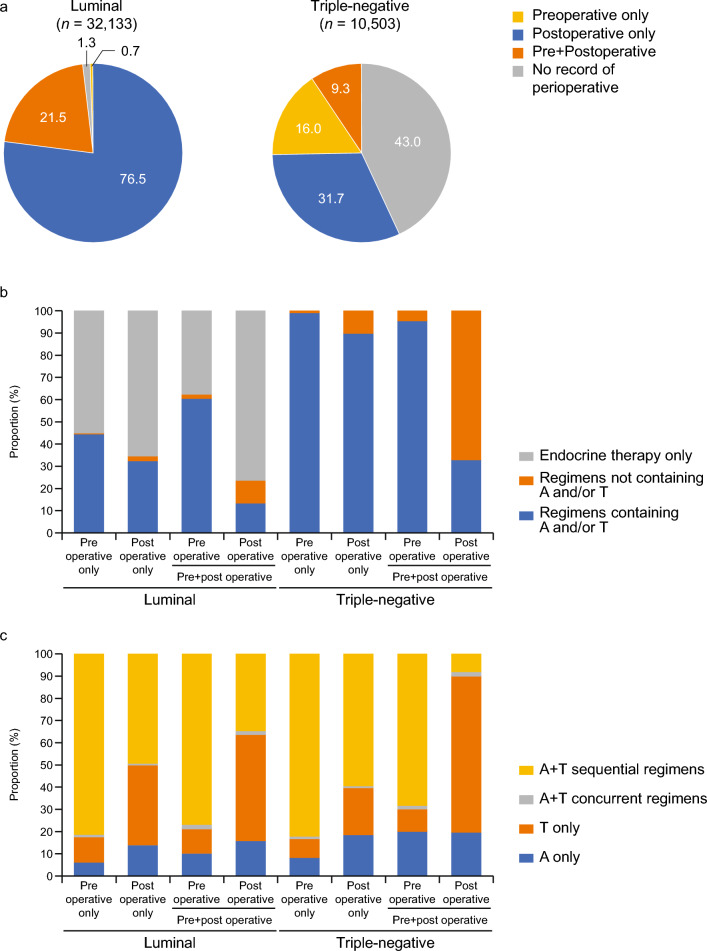
Treatment pattern in perioperative anti-tumor therapy. **a** Proportion of patients for each perioperative therapy. **b** Proportion of patients with regimens during the perioperative period. **c** Proportion of patients with regimens including anthracycline, taxane, or both during the perioperative period. *A* anthracycline, *T* taxane

In the luminal-type group (*n* = 32,133), postoperative therapy alone was the most common treatment (24,590 patients [76.5%]). This was followed by 6917 patients (21.5%) who received both pre- and postoperative therapies and 219 patients (0.7%) who received only preoperative therapy (Fig. 3a). A total of 18,200 patients (56.6%) received endocrine therapy alone. Among the patients who received only preoperative therapy (*n* = 219), 121 (55.3%) patients were treated with endocrine therapy alone, and among those who received only postoperative therapy (*n* = 24,590), 16,150 (65.7%) patients were treated with endocrine therapy alone. In both pre- and postoperative therapy (*n* = 6917) chemotherapy regimens, anthracycline, taxane, or both tended to be more likely to be received as preoperative therapy than other regimens (preoperative in preoperative + postoperative: 4186 patients [60.5%]) (Fig. 3b).

Among chemotherapy regimens including anthracycline, taxane, or both, anthracycline + taxane sequential regimens tended to be used as preoperative or postoperative therapy (preoperative only: 79/97 patients [81.4%], postoperative only: 3887/7895 patients [49.2%], and preoperative in preoperative + postoperative: 3216/4186 patients [76.8%]). When a regimen containing anthracycline, taxane, or both was selected as postoperative therapy in patients with both pre- and postoperative therapies (*n* = 6917), taxane only or anthracycline + taxane sequential regimens were more likely to be received as treatment (postoperative in preoperative + postoperative: taxane only, 434/906 patients [47.9%] and anthracycline + taxane sequential, 313/906 patients [34.5%]) (Fig. 3c).

In the triple-negative group (*n* = 10,503), 4516 patients (43.0%) had no record of either pre- or postoperative therapy (Fig. 3a). In total, 3327/10,503 patients (31.7%) received only postoperative therapy, followed by 1683/10,503 patients (16.0%) receiving only preoperative therapy, and 977/10,503 patients (9.3%) received both pre- and postoperative therapies. Regarding treatment patterns, chemotherapy regimens including anthracycline, taxane, or both were administered in about 90% or more of cases where pre- or postoperative therapy was received (preoperative only: 1667/1683 patients [99.0%], postoperative only: 2982/3327 patients [89.6%], and preoperative in preoperative + postoperative: 930/977 patients [95.2%]), whereas a regimen not containing anthracycline, taxane, or both was selected as postoperative therapy: 658/977 (67.3%) in triple-negative patients (Fig. 3b). When both pre- and postoperative therapies were received (*n* = 977), chemotherapy regimens without anthracycline or taxane were received as postoperative therapy in 658/977 patients (67.3%). Among patients receiving chemotherapy with anthracycline, taxane, or both, anthracycline + taxane sequential regimens were more likely to be selected as pre- or postoperative therapies than other regimens containing anthracycline, taxane, or both (preoperative only: 1369/1667 patients [82.1%]; postoperative only: 1766/2982 patients [59.2%]; and preoperative in preoperative + postoperative: 635/930 patients [68.3%]) (Fig. 3c). When regimens containing anthracycline, taxane, or both were used as postoperative therapy in patients with both pre- and postoperative therapies (319/977 [32.7%]), a regimen containing taxane only was received by a higher proportion of patients (224/319 [70.2%]).

### Factors contributing to the prescription of regimens, including anthracycline, taxane, or both

Table 2 summarizes the ORs for receiving regimens containing anthracycline, taxane, or both. In the luminal-type group, patients ≥ 40 years of age were less likely to receive chemotherapy regimens including anthracycline, taxane, or both than patients < 40 years of age (number of patients, [%], OR [95% CI]  < 40 years, 968/1381 patients [70.1%]; 40–49 years, 3733/6383, [58.5%], 0.62 [0.54–0.70]; 50–59 years, 3195/5624, [56.8%], 0.56 [0.49–0.63]; 60–69 years, 3417/7605, [44.9%], 0.33 [0.29–0.38]; and ≥ 70 years, 1460/10733, [13.6%], 0.06 [0.05–0.06]).

**Table 2:** Odds ratios for patients receiving regimens containing anthracycline and/or taxane in pre- or postoperative therapy

Parameter	Category	Luminal						Triple-negative					
		Regimens containing A and/or T *n* (%)	Regimens not containing A and/or T *n* (%)	Univariate analysis		Multivariate analysis		Regimens containing A and/or T *n* (%)	Regimens not containing A and/or T *n* (%)	Univariate analysis		Multivariate analysis	
				Estimate (odds ratio)	95% CI	Estimate (adjusted odds ratio)	95% CI			Estimate (odds ratio)	95% CI	Estimate (adjusted odds ratio)	95% CI
Age, years (reference: < 40)	< 40	968 (70.1)	413 (29.9)	–	–	–	–	453 (98.7)	6 (1.3)	–	–	–	–
	40–49	3733 (58.5)	2650 (41.5)	0.60	0.53–0.68	0.62	0.54–0.70	1092 (99.0)	11 (1.0)	1.31	0.48–3.58	1.31	0.48–3.57
	50–59	3195 (56.8)	2429 (43.2)	0.56	0.49–0.64	0.56	0.49–0.63	1343 (98.6)	19 (1.4)	0.94	0.37–2.36	0.95	0.38–2.40
	60–69	3417 (44.9)	4188 (55.1)	0.35	0.31–0.39	0.33	0.29–0.38	1644 (95.7)	74 (4.3)	0.29	0.13–0.68	0.30	0.13–0.69
	≥ 70	1460 (13.6)	9273 (86.4)	0.07	0.06–0.08	0.06	0.05–0.06	1062 (79.0)	283 (21.0)	0.05	0.02–0.11	0.05	0.02–0.12
Hospital capacity, beds (reference: < 200)	< 200	322 (31.8)	692 (68.2)	–	–	–	–	111 (84.1)	21 (15.9)	–	–	–	–
	200–499	6585 (39.2)	10,196 (60.8)	1.39	1.21–1.59	1.02	0.87–1.19	2833 (92.8)	219 (7.2)	2.45	1.50–3.98	1.77	1.02–3.09
	≥ 500	5866 (42.1)	8065 (57.9)	1.56	1.36–1.79	0.93	0.79–1.10	2650 (94.5)	153 (5.5)	3.28	2.00–5.37	2.00	1.10–3.64
Cancer therapeutic facility status (reference: Yes)	Yes	10,729 (42.2)	14,719 (57.8)	–	–	–	–	4676 (94.1)	295 (5.9)	–	–	–	–
	No	2044 (32.6)	4234 (67.4)	0.66	0.62–0.70	0.65	0.60–0.70	918 (90.4)	98 (9.6)	0.59	0.47–0.75	0.74	0.55–0.99
Clinical stage (reference: III)	II	9467 (35.9)	16,874 (64.1)	0.35	0.33–0.37	0.24	0.22–0.25	4256 (93.5)	298 (6.5)	1.01	0.80–1.29	0.95	0.74–1.23
	III	3306 (61.4)	2079 (38.6)	–	–	–	–	1338 (93.4)	95 (6.6)	–	–	–	–

This analysis was performed for patients with no missing data for all parameters

*A* anthracycline, *CI* confidence interval, *T* taxane

Patients treated at facilities that were not cancer treatment centers (2044/6278 patients [32.6%]) tended to receive regimens containing anthracycline, taxane, or both less often than those in specialized cancer treatment centers (10,729/25,448 patients [42.2%]) (OR [95% CI] 0.65 [0.60–0.70]). Patients with clinical stage II (9467/26,341 patients [35.9%]) tended to be prescribed regimens containing anthracycline, taxane, or both less often than those with clinical stage III (3306/5385 patients [61.4%]) (OR [95% CI] 0.24 [0.22–0.25]).

In the triple-negative group, patients aged 60–69 years (1644/1718 [95.7%]) and ≥ 70 years (1062/1345 [79.0%]) tended to be prescribed regimens containing anthracycline, taxane, or both less often than those aged < 40 years (453/459 [98.7%]) (OR [95% CI] 0.30 [0.13–0.69] and 0.05 [0.02–0.12], respectively). In addition, patients treated at hospitals with capacities of < 200 beds (111/132 patients [84.1%]) less often received regimens including anthracycline, taxane, or both, compared with those treated at hospitals with capacities of 200–499 (2833/3052 patients [92.8%]) and ≥ 500 beds (2650/2803 patients [94.5%]) (ORs [95% CI] 1.77 [1.02–3.09] and 2.00 [1.10–3.64], respectively). Additionally, regimens containing anthracycline, taxane, or both were less likely to be administered in facilities that were not cancer treatment centers (918/1016 patients [90.4%]) than in those specializing in cancer treatment (4676/4971 patients [94.1%]) (OR [95% CI] 0.74 [0.55–0.99]).

### Treatment duration for each treatment regimen

**Fig. 4:**
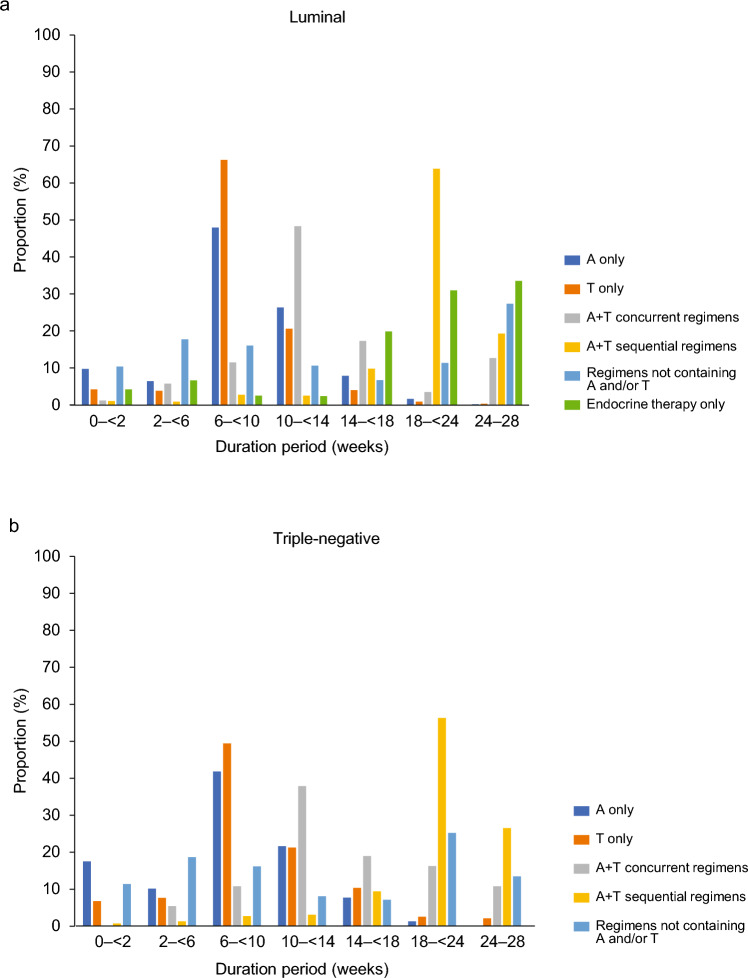
Proportion of patients in each category of treatment duration. Treatment duration was stratified into 0– < 2, 2– < 6, 6– < 10, 10– < 14, 14– < 18, 18– < 24, and 24–28 weeks from the first prescription date of initial adjuvant therapy to the date of death, the date of loss to follow-up of treatment regimen duration, the date of censoring at 30 September 2021, or the date of the prescription after 24 weeks, whichever occurred first. **a** Luminal-type, and **b** triple-negative group. *A* anthracycline, *T* taxane

For regimens with anthracycline only and taxane only in the luminal-type group, the most common treatment duration was 6– < 10 weeks (anthracycline only, 592/1235 patients [47.9%]; taxane only, 2171/3279 patients [66.2%]). The most common duration for concurrent anthracycline + taxane treatment was 10– < 14 weeks (42/87 patients [48.3%]). For anthracycline + taxane sequential regimens, the most common treatment duration was 18– < 24 weeks (2679/4200 patients [63.8%]). In regimens not including anthracycline or taxane, 226/1275 patients (17.7%) had treatment durations of 2– < 6 weeks and 204/1275 patients (16.0%) had treatment duration of 6– < 10 weeks. When only endocrine therapy was received, 6636/21,431 patients (31.0%) and 7177/21,431 patients (33.5%) had treatment durations of 18– < 24 weeks and 24–28 weeks, respectively (Fig. 4a).

For chemotherapy regimens with anthracycline only and taxane only in the triple-negative group, the most common treatment duration was 6– < 10 weeks (anthracycline only, 256/612 [41.8%] patients; taxane only, 425/861 patients [49.4%]). The most common treatment duration for the anthracycline + taxane concurrent regimen was 10– < 14 weeks (14/37 patients [37.8%]). For the anthracycline + taxane sequential regimen, 18– < 24 weeks treatment durations were the most common (1009/1791 patients [56.3%]). In regimens not including anthracycline or taxane, 253/1003 patients (25.2%) had treatment durations of 18– < 24 weeks, 187/1003 (18.6%) had treatment durations of 2– < 6 weeks, and 162/1003 (16.2%) had treatment durations of 6– < 10 weeks (Fig. 4b).

## Discussion

We conducted a descriptive study to clarify the actual patterns of perioperative chemotherapy regimens including anthracycline, taxane, or both in T ≥ 2, N+ , high-risk, early-stage, breast cancer patients not receiving anti-HER2 therapy. In the luminal-type and triple-negative groups, 98.7% and 57.0% of patients, respectively, received perioperative therapy, and of those most (94.4% and 93.4%, respectively) were treated with regimens including anthracyclines and/or taxane. These results indicate that patients who received chemotherapy had a prioritized standard of care regimen. According to the results from the National Clinical Database in Japan for breast cancer patients who had surgery in 2018 or non-surgical patients who began treatment in 2018 regardless of clinical stage [4], 9551/12,846 patients (74.3%) who received preoperative treatment and 18,989/71,278 patients (26.6%) who received postoperative treatment received chemotherapy, which is consistent with the data observed in our study targeting higher risk patients. However, our data revealed that only 12,773/32,133 patients (67.8%) in the luminal-type group and 5594/10,503 (53.3%) in the triple-negative group received chemotherapy regimens including perioperative anthracycline and/or taxane, suggesting that a substantial number of patients with T ≥ 2 and N+ breast cancer and considered to be at high risk of recurrence may not have received sufficient anthracycline/taxane treatment to prevent such recurrence. For patients with T ≥ 2 and N+, considered to be at high risk of recurrence, we also clarified the factors associated with selecting chemotherapy regimens, including anthracycline, taxane, or both. Regimens with anthracycline, taxane or both were particularly selected for younger patients at a more advanced clinical stage. In this study, the trend to avoid anthracycline treatment in older patients suggests that such patients may actually be undertreated, despite the importance of receiving perioperative therapy to prevent recurrence in early-stage breast cancer without distant metastasis.

This study also showed that the proportions of patients receiving anthracycline + taxane treatment were low in facilities with smaller capacities and hospitals without specialized cancer centers. Treatment choices may differ depending on facility function and capacity, which may represent a clinical barrier for patients to access optimal treatment. For patients at high risk of recurrence, a medical environment should be developed to provide them with such life-saving access.

The most common treatment duration was 18– < 24 weeks for the anthracycline + taxane sequential regimen and 6– < 10 weeks for the anthracycline only and taxane only regimens in both groups. This is comparable to the treatment durations of chemotherapy regimens in the Breast Cancer Clinical Guideline 2022 [28]. However, the duration of treatment with endocrine therapy might be estimated as shorter than the expected duration of 5–10 years because of the short common duration (28 weeks) of observation in this study, which would be insufficient to capture the full duration of endocrine therapy.

### Limitations and strengths

The database utilized in this study is based on the hospital-based claims data collected from acute care hospitals, which may not include all breast cancer patients in Japan. Therefore, the findings of this research may not be applicable to the broader medical landscape in Japan. Data used in this study were not collected for specific research purposes. Thus, it was impossible to validate all confounding factors, such as performance status, clinical laboratory data, and pathological classification, which could not be extracted from the database. Differences in facility and physician preferences for treatment are important confounding factors that affect treatment choices, but this information could not be collected in this study. Additionally, cancer stage data and TNM classification were only available for hospitalized patients. Because of the database limitations, inter-facility patient tracking was not possible, and data on pre- and postoperative therapies at facilities different from those where surgery was performed were not recorded.

The database does not include complete medical records and does not capture the patients’ pathological and molecular profiles, and classification into study groups was based on treatment patterns rather than molecular expression (luminal-type/triple-negative groups). Accordingly, elderly patients or patients who could not receive anti-HER2 therapy or endocrine therapy because of comorbidities may be mistakenly regarded as triple-negative here, even if they were pathologically HER2+ or HR+. This may explain why there were many untreated patients in the triple-negative group. The number of untreated patients included in the triple-negative group in this study may have been higher than the number of the actual pathologically triple-negative patients who had not received chemotherapy. Therefore, our interpretations of treatment reality by subtype classification are limited.

## Conclusions

This is the first study to investigate the characteristics and treatment pattern of patients who have been clinically selected to receive anthracyclines and/or taxanes, focusing on Japanese patients with HER2− early-stage breast cancer who are at high risk of recurrence. We examined how pre- and postoperative therapy was prescribed and found that for prevention of recurrence, anthracycline and/or taxane may not have been prescribed adequately as perioperative treatment, and older patients in earlier clinical stages tended not to receive these as part of their treatment regimens. This study also clarified that perioperative treatment options seem to differ depending on the capacity and function of the facility where patients receive treatment and has shed light on the challenges of breast cancer treatment in Japan. In the future, it is necessary to assess the impact and causes of the medical disparities revealed in this study and promote the improvement of the medical environment and the equalization of treatment for breast cancer in Japan.

### Supplementary Information

Below is the link to the electronic supplementary material.

Supplementary file1 (PDF 237 KB)

## Data Availability

The data included in this manuscript were used under contract with the supplier (Medical Data Vision Co., Ltd.) and cannot be freely distributed by the authors.
