# Supplementary material for: Real-world data of HER2-negative early breast cancer patients treated with anthracycline and/or taxane regimens in Japan
Source: Breast Cancer. 2024 Apr 29;31(4):581–92. doi: 10.1007/s12282-024-01572-8 (PMC11194198; doi:10.1007/s12282-024-01572-8)
Supplement: Supplementary file 1 — Supplementary file1 (PDF 237 KB) [file 12282_2024_1572_MOESM1_ESM.pdf]

Real-world data of HER2-negative early breast cancer patients treated with anthracycline and/or taxane regimens in Japan

Breast Cancer

Akihiko Shimomura[1], Yasuaki Sagara[2], Ryo Koto[3], Masakazu Fujiwara[3], Yuka Kanemura[3], Hiroshi Kitagawa[3], Shigehira Saji[4]

[1]Department of Breast and Medical Oncology, National Center for Global Health and Medicine, Tokyo, Japan.

[2]Department of Breast and Thyroid Surgical Oncology, Social Medical Corporation Hakuaikai Sagara Hospital, Kagoshima, Japan.

[3]Medical Department, AstraZeneca K.K., Osaka, Japan.

[4]Department of Medical Oncology, Fukushima Medical University, Fukushima, Japan.

Corresponding author

Professor Shigehira Saji, Department of Medical Oncology, Fukushima Medical University, 1 Hikarigaoka, Fukushima City, Fukushima, 960-1295, Japan.

E-mail: ss-saji@wa2.so-net.ne.jp

Online Resource 1 List of ICD-10 codes for breast cancer diagnosis

Disease Name	ICD-10 code
Mammary Paget's disease, Nipple Breast Cancer, Areolar Breast Cancer	C500
Central Breast Cancer	C501
Upper-inner Quadrant Breast Cancer	C502
Lower-inner Quadrant Breast Cancer	C503
Upper-outer Quadrant Breast Cancer	C504
Lower-outer Quadrant Breast Cancer	C505
Mammary Gland Axillary-tail Breast Cancer, Axillary Breast Cancer	C506
Overlapping Breast Cancer, Ectopic Breast Cancer	C508
Postoperative Breast Cancer, Breast Cancer, Breast Cancer Recrudescence, Malignant Mammary Gland Tumor, Malignant Breast Tumor, Breast Sarcoma, Inflammatory Breast Cancer, Malignant Phyllodes Tumor, Advanced Breast Cancer, Breast Cancer - HER2 Overexpression, Breast Liposarcoma, Breast Angiosarcoma, Breast Fibrosarcoma, Invasive Ductal Carcinoma, Multiple Breast Cancer, Papillotubular Carcinoma, Mammary Gland Scirrhous Carcinoma, Mammary Gland Solid-tubular Carcinoma, HER2 Positive Breast Cancer, Breast Cancer Local Recurrence, Breast Cancer Postoperative Recurrence on Chest Wall	C509

ICD-10 International Classification of Diseases, 10th revision.

Online Resource 2 List of surgery receipt codes for primary breast cancer

**Name of classification**	**Medical act**	**Receipt code**
Partial mastectomy	Partial mastectomy	150274610
Mastectomy	Mastectomy	150121410
	Mastectomy (Hereditary Breast and Ovarian Cancer patients)	150413710
Destruction of breast lesion	Destruction of breast lesion	150121550
Mammary gland malignancy surgery	Simple mastectomy (total mastectomy)	150121610
	Simple bilateral mastectomy with lymph node radical excision	150121710
	Lumpectomy with axillary dissection	150121810
	Extended radical bilateral mastectomy	150121910
	Partial mastectomy (with axillary lymph node radical excision)	150262710
	Partial mastectomy (without axillary lymph node radical excision)	150303110
	Mastectomy (without axillary dissection)	150316510
	Nipple-sparing mastectomy (without axillary dissection)	150386410
	Nipple-sparing mastectomy (with axillary dissection)	150386510
	Mammary gland malignancy surgery and both sides of lymph node dissection	150122150

Online Resource 3 List of drug therapy receipt codes

Type	Generic name	Receipt code
Antineoplastic drug	Trastuzumab	640451013, 620001938, 622069901, 622069801
	Pertuzumab	622255101
	Trastuzumab emtansine	622264501, 622628901, 622659701, 622629001, 622659801, 622264401, 622630801, 622679301, 622630701, 622679201
	Trastuzumab deruxtecan	629907101
	Lapatinib	621911601
	Doxorubicin	620004851, 620003675, 622014001, 646180002, 621983201, 621983301, 621995301, 621995401
	Epirubicin	620003790, 620003791, 620003792, 620003793, 620002978, 620002979, 640433066, 646180019, 620007224, 620007225, 620008174, 620008175, 620009523, 620009526, 621966401, 621966601, 622760200, 622760300, 622760400, 620002905, 620002906, 620006694, 620006695, 620009524, 620009525, 620009527, 621966501, 621966701, 622246601, 622246701
	Paclitaxel	620003751, 620003752, 640412113, 640432023, 620004170, 620004171, 620005688, 620005689, 620005690, 622082001, 622082101, 622259101, 622259201, 622375001, 622375101, 622760500, 622760600, 622760700, 622009101, 622009201, 622009102, 622009202
	Docetaxel	620919801, 620919901, 640411025, 640411026, 622068501, 622068601, 622215301, 622215401, 622272001, 622272101, 622285201, 622285301, 622285401, 622294901, 622295001, 622295501, 622295601, 622354801, 622354901, 622356401, 622356501, 622408501, 622408601, 622429301, 622429401, 622435002, 622435102, 622231801, 622231901, 622417601, 622417701, 622290401, 622290501, 622283101, 622283201
	Nab-paclitaxel	621970101
	Atezolizumab	622594601, 629900601
	Bevacizumab	620004872, 620004873, 629904901, 629905001, 629905901, 629906001
	Pembrolizumab	622515801, 622515701
	Eribulin	622085201
	Vinorelbine	621954401, 621954501, 640432004, 640432005
	Gemcitabine	640454012, 640454013, 640432018, 640432019, 621970201, 621970202, 621970301, 621970302, 621973401, 621973501, 622019601, 622019701, 622028601, 622028701, 622062103, 622062105, 622062203, 622062205, 622098901, 622099001, 622202401, 622202501, 622393001, 622393101, 622460401, 622460501, 622487701, 622487801, 621994401, 621994501, 622062101, 622062201, 622272801, 622272901
	Capecitabine	610470009, 622656401, 622674301, 622677701, 622679001, 622695801, 622700101
	Olaparib	622606901, 622607001

	Carboplatin	620004732, 620004733, 620004734, 644210074, 644210075, 644210076, 644210077, 620000216, 620000217, 620002932, 620004117, 620004118, 620004119, 620004120, 620004121, 620004122, 620007254, 620007255, 620007256, 621754502, 621754602, 621754702, 622098103, 622098203, 622098303, 622761100, 622761200, 622761300, 622882601, 622882701, 622882801, 620002929, 620002930, 620002931, 620004114, 620004115, 620004116, 622098101, 622098201, 622098301
	Everolimus	621980901, 622216801, 622226301, 622226401
	Irinotecan	620007257, 620007258, 644290006, 644290007, 620919501, 620919701, 644290008, 644290009, 622808601, 620009515, 620009516, 620009519, 620009520, 621900302, 621900402, 622019401, 622019501, 622059701, 622059801, 622230201, 622230301, 622236901, 622237001, 622258901, 622259001, 622470401, 622470501, 620009517, 620009521, 622091101, 622091201, 620009518, 620009522
	Tegafur, gimeracil, and oteracil combination	622254901, 622255001, 622434701, 622434801, 622487301, 622487401, 622275701, 622275801, 622256001, 622256101, 622430801, 622430901, 622497901, 622498001, 622397101, 622397201, 622397301, 622397401, 622537501, 622537601, 620009353, 620009354, 620915501, 620915601, 622243001, 622243101, 610421353, 610421354, 622294601, 622294701
	Abemaciclib	622653801, 622653901, 622654001
Endocrine therapy	Fulvestrant	622101401
	Goserelin	640443027, 640462004, 642490105
	Leuprorelin	620555101, 620555201, 620555301, 620555401, 621495301, 622444901, 640406224, 640432015, 640432016, 640462036, 642490119, 622266501, 622266601, 622298301, 622298401
	Tamoxifen	620003593, 620003594, 614210059, 614210149, 620001885, 620920504, 620921005, 620921501, 620921701, 620921905, 622041701, 622053001, 622075101, 622317900, 622671201, 622671301, 614210152, 620921003, 620921903
	Anastrozole	620003507, 610443065, 622180501, 622195001, 622195501, 622198501, 622204401, 622208401, 622208701, 622211201, 622213401, 622215501, 622218301, 622238501, 622671101, 622689100, 622213701, 622222601, 622309400
	Exemestane	610462026, 622115801, 622118801, 622158301
	Letrozole	620003467, 622411401, 622412801, 622413201, 622417401, 622418401, 622418402, 622420001, 622422101, 622427401, 622427901, 622429201, 622429901, 622431001, 622432001, 622433901, 622435201, 622436701, 622438901, 622475600
	Toremifene	610407022, 610407023, 620004006, 622169001, 622742600, 622742700
	Medroxyprogesterone	612470030, 620008693, 612470052, 620537901, 612470038, 612470053, 620537802, 620538201, 620538401, 621285301, 622736700, 620538001

Online Resource 4 Proportion of patients for each pre- and postoperative therapy treatment pattern

		Luminal (*n* = 41,733)					Triple-negative (*n* = 37,044)				
		Preoperative only	Postoperative only	Pre+postoperative		No pre+postoperative	Preoperative only	Postoperative only	Pre+postoperative		No pre+postoperative
				Preoperative	Postoperative				Preoperative	Postoperative	
Total	*n*, %	400 (100.0)	32,300 (100.0)	8,021 (100.0)	8,021 (100.0)	1,012 (100.0)	2,463 (100.0)	6,049 (100.0)	1,254 (100.0)	1,254 (100.0)	27,278 (100.0)
Regimens containing A and/or T	*n*, %	119 (29.8)	8,567 (26.5)	4,522 (56.4)	994 (12.4)	---	2,386 (96.9)	5,321 (88.0)	1,141 (91.0)	422 (33.7)	---
	95% CI	[24.60–35.48]	[25.94–27.12]	[55.05–57.70]	[11.54–13.30]	---	[95.99–97.57]	[87.00–88.87]	[89.01–92.64]	[30.73–36.70]	---
Regimens not containing A and/or T	*n*, %	4 (1.0)	657 (2.0)	175 (2.2)	831 (10.4)	---	77 (3.1)	728 (12.0)	113 (9.0)	832 (66.3)	---
	95% CI	[0.32–3.06]	[1.85–2.23]	[1.82–2.61]	[9.57–11.20]	---	[2.43–4.01]	[11.13–13.00]	[7.36–10.99]	[63.30–69.27]	---
Endocrine therapy only	*n*, %	277 (69.3)	23,076 (71.4)	3,324 (41.4)	6,196 (77.2)	---	---	---	---	---	---
	95% CI	[63.49–74.47]	[70.84–72.04]	[40.13–42.76]	[76.11–78.35]	---	---	---	---	---	---
No chemotherapy and no endocrine therapy	*n*, %	---	---	---	---	1,012 (100.0)	---	---	---	---	27,278 (100.0)
	95% CI	---	---	---	---	[99.62–100.00]	---	---	---	---	[99.99–100.00]

A anthracycline, *CI* confidence interval, *T* taxane

Online Resource 5 Patient characteristics in the luminal-type group

			Luminal-type								
				Regimens containing A and/or T							
			Overall	All	A only	T only	A+T concurrent regimens	A+T sequential regimens	Regimens not containing A and/or T	Endocrine therapy only	No chemotherapy and no endocrine therapy
Number of patients (*N*)		42,636	32,133	12,773	1,510	3,670	162	7,431	753	18,200	407
Age, years	Median	64	63	54	58	57	53	53	68	69	66
	Min	19	19	19	28	19	31	21	28	23	26
	Max	100	100	87	86	87	81	86	94	100	97
	<40	2,029 (4.8)	1,409 (4.4)	968 (7.6)	83 (5.5)	191 (5.2)	12 (7.4)	682 (9.2)	12 (1.6)	401 (2.2)	28 (6.9)
	40–49	8,078 (18.9)	6,469 (20.1)	3,733 (29.2)	377 (25.0)	984 (26.8)	54 (33.3)	2,318 (31.2)	94 (12.5)	2,556 (14.0)	86 (21.1)
	50–59	7,546 (17.7)	5,672 (17.7)	3,195 (25.0)	356 (23.6)	848 (23.1)	40 (24.7)	1,951 (26.3)	107 (14.2)	2,322 (12.8)	48 (11.8)
	60–69	10,095 (23.7)	7,680 (23.9)	3,417 (26.8)	462 (30.6)	1,068 (29.1)	40 (24.7)	1,847 (24.9)	202 (26.8)	3,986 (21.9)	75 (18.4)
	≥70	14,888 (34.9)	10,903 (33.9)	1,460 (11.4)	232 (15.4)	579 (15.8)	16 (9.9)	633 (8.5)	338 (44.9)	8,935 (49.1)	170 (41.8)
Sex	Male	307 (0.7)	287 (0.9)	60 (0.5)	14 (0.9)	18 (0.5)	1 (0.6)	27 (0.4)	9 (1.2)	213 (1.2)	5 (1.2)
	Female	42,329 (99.3)	31,846 (99.1)	12,713 (99.5)	1,496 (99.1)	3,652 (99.5)	161 (99.4)	7,404 (99.6)	744 (98.8)	17,987 (98.8)	402 (98.8)
T factor	0	38 (0.1)	24 (0.1)	17 (0.1)	2 (0.1)	3 (0.1)	0	12 (0.2)	1 (0.1)	6 (0.0)	0
	1	4,967 (11.6)	4,051 (12.6)	1,923 (15.1)	245 (16.2)	551 (15.0)	21 (13.0)	1,106 (14.9)	100 (13.3)	1,981 (10.9)	47 (11.5)
	2	30,999 (72.7)	23,266 (72.4)	8,379 (65.6)	985 (65.2)	2,627 (71.6)	107 (66.0)	4,660 (62.7)	494 (65.6)	14,095 (77.4)	298 (73.2)
	3	2,868 (6.7)	1,972 (6.1)	1,059 (8.3)	115 (7.6)	193 (5.3)	14 (8.6)	737 (9.9)	65 (8.6)	819 (4.5)	29 (7.1)
	4	3,764 (8.8)	2,820 (8.8)	1,395 (10.9)	163 (10.8)	296 (8.1)	20 (12.3)	916 (12.3)	93 (12.4)	1,299 (7.1)	33 (8.1)
N factor	0	23,674 (55.5)	17,866 (55.6)	4,439 (34.8)	575 (38.1)	1,915 (52.2)	54 (33.3)	1,895 (25.5)	372 (49.4)	12,821 (70.4)	234 (57.5)

	1	15,368 (36.0)	11,823 (36.8)	6,521 (51.1)	747 (49.5)	1,483 (40.4)	85 (52.5)	4,206 (56.6)	318 (42.2)	4,842 (26.6)	142 (34.9)
	2	2,141 (5.0)	1,520 (4.7)	1,041 (8.2)	125 (8.3)	164 (4.5)	13 (8.0)	739 (9.9)	41 (5.4)	413 (2.3)	25 (6.1)
	3	1,453 (3.4)	924 (2.9)	772 (6.0)	63 (4.2)	108 (2.9)	10 (6.2)	591 (8.0)	22 (2.9)	124 (0.7)	6 (1.5)
M factor	0	42,636 (100.0)	32,133 (100.0)	12,773 (100.0)	1,510 (100.0)	3,670 (100.0)	162 (100.0)	7,431 (100.0)	753 (100.0)	18,200 (100.0)	407 (100.0)
	1	0	0	0	0	0	0	0	0	0	0
Clinical stage	I	0	0	0	0	0	0	0	0	0	0
	II	35,042 (82.2)	26,679 (83.0)	9,467 (74.1)	1,156 (76.6)	3,104 (84.6)	118 (72.8)	5,089 (68.5)	588 (78.1)	16,286 (89.5)	338 (83.0)
	III	7,594 (17.8)	5,454 (17.0)	3,306 (25.9)	354 (23.4)	566 (15.4)	44 (27.2)	2,342 (31.5)	165 (21.9)	1,914 (10.5)	69 (17.0)
Hospital capacity, beds	<200	1,371 (3.2)	1,024 (3.2)	322 (2.5)	66 (4.4)	77 (2.1)	3 (1.9)	176 (2.4)	37 (4.9)	655 (3.6)	10 (2.5)
	200–499	22,550 (52.9)	17,031 (53.0)	6,585 (51.6)	872 (57.7)	1,930 (52.6)	92 (56.8)	3,691 (49.7)	401 (53.3)	9,795 (53.8)	250 (61.4)
	≥500	18,715 (43.9)	14,078 (43.8)	5,866 (45.9)	572 (37.9)	1,663 (45.3)	67 (41.4)	3,564 (48.0)	315 (41.8)	7,750 (42.6)	147 (36.1)
Cancer therapeutic facility status	Yes	34,241 (80.3)	25,747 (80.1)	10,729 (84.0)	1,176 (77.9)	3,130 (85.3)	120 (74.1)	6,303 (84.8)	554 (73.6)	14,165 (77.8)	299 (73.5)
	No	8,395 (19.7)	6,386 (19.9)	2,044 (16.0)	334 (22.1)	540 (14.7)	42 (25.9)	1,128 (15.2)	199 (26.4)	4,035 (22.2)	108 (26.5)
Follow-up period, weeks	Median	157.43	168.29	181.43	206.00	192.00	209.29	169.86	165.00	159.29	189.86
	Min	4.3	4.3	4.3	6.7	6.3	9.4	4.3	5.9	4.3	28.0
	Max	659.4	659.4	659.4	659.4	584.9	646.7	653.1	647.4	622.1	576.6

Data are *n* (%) unless otherwise stated.

A anthracycline, *M* factor metastasis, *Max* maximum, *Min* minimum, *N* factor lymph node, *T* taxane, *T* factor tumor size

Online Resource 6 Patient characteristics in the triple-negative group

		Triple-negative							
			Regimens containing A and/or T					Regimens not containing A and/or T	No chemotherapy and no endocrine therapy
		Overall	All	A only	T only	A+T concurrent regimens	A+T sequential regimens		
Number of patients (*N*)		10,503	5,594	787	1,046	70	3,691	393	4,516
Age, years	Median	65	59	63	63	56	57	76	74
	Min	23	23	28	26	32	23	31	24
	Max	100	95	95	87	85	85	92	100
	<40	620 (5.9)	453 (8.1)	47 (6.0)	59 (5.6)	8 (11.4)	339 (9.2)	6 (1.5)	161 (3.6)
	40–49	1,609 (15.3)	1,092 (19.5)	122 (15.5)	166 (15.9)	16 (22.9)	788 (21.3)	11 (2.8)	506 (11.2)
	50–59	1,874 (17.8)	1,343 (24.0)	153 (19.4)	200 (19.1)	19 (27.1)	971 (26.3)	19 (4.8)	512 (11.3)
	60-69	2,415 (23.0)	1,644 (29.4)	254 (32.3)	293 (28.0)	20 (28.6)	1,077 (29.2)	74 (18.8)	697 (15.4)
	≥70	3,985 (37.9)	1,062 (19.0)	211 (26.8)	328 (31.4)	7 (10.0)	516 (14.0)	283 (72.0)	2,640 (58.5)
Sex	Male	20 (0.2)	3 (0.1)	0	1 (0.1)	0	2 (0.1)	1 (0.3)	16 (0.4)
	Female	10,483 (99.8)	5,591 (99.9)	787 (100.0)	1,045 (99.9)	70 (100.0)	3,689 (99.9)	392 (99.7)	4,500 (99.6)
T factor	0	14 (0.1)	13 (0.2)	3 (0.4)	2 (0.2)	0	8 (0.2)	1 (0.3)	0
	1	916 (8.7)	595 (10.6)	85 (10.8)	87 (8.3)	5 (7.1)	418 (11.3)	31 (7.9)	290 (6.4)
	2	7,733 (73.6)	4,014 (71.8)	558 (70.9)	756 (72.3)	43 (61.4)	2,657 (72.0)	279 (71.0)	3,440 (76.2)
	3	896 (8.5)	479 (8.6)	71 (9.0)	87 (8.3)	11 (15.7)	310 (8.4)	26 (6.6)	391 (8.7)
	4	944 (9.0)	493 (8.8)	70 (8.9)	114 (10.9)	11 (15.7)	298 (8.1)	56 (14.2)	395 (8.7)
N factor	0	5,808 (55.3)	2,523 (45.1)	394 (50.1)	534 (51.1)	28 (40.0)	1,567 (42.5)	200 (50.9)	3,085 (68.3)
	1	3,545 (33.8)	2,228 (39.8)	294 (37.4)	359 (34.3)	31 (44.3)	1,544 (41.8)	149 (37.9)	1,168 (25.9)
	2	621 (5.9)	440 (7.9)	54 (6.9)	76 (7.3)	8 (11.4)	302 (8.2)	23 (5.9)	158 (3.5)
	3	529 (5.0)	403 (7.2)	45 (5.7)	77 (7.4)	3 (4.3)	278 (7.5)	21 (5.3)	105 (2.3)

M factor	0	10,503 (100.0)	5,594 (100.0)	787 (100.0)	1,046 (100.0)	70 (100.0)	3,691 (100.0)	393 (100.0)	4,516 (100.0)
	1	0	0	0	0	0	0	0	0
Clinical stage	I	0	0	0	0	0	0	0	0
	II	8,363 (79.6)	4,256 (76.1)	618 (78.5)	795 (76.0)	44 (62.9)	2,799 (75.8)	298 (75.8)	3,809 (84.3)
	III	2,140 (20.4)	1,338 (23.9)	169 (21.5)	251 (24.0)	26 (37.1)	892 (24.2)	95 (24.2)	707 (15.7)
Hospital capacity, beds	<200	347 (3.3)	111 (2.0)	26 (3.3)	18 (1.7)	2 (2.9)	65 (1.8)	21 (5.3)	215 (4.8)
	200–499	5,519 (52.5)	2,833 (50.6)	444 (56.4)	556 (53.2)	35 (50.0)	1,798 (48.7)	219 (55.7)	2,467 (54.6)
	≥500	4,637 (44.1)	2,650 (47.4)	317 (40.3)	472 (45.1)	33 (47.1)	1,828 (49.5)	153 (38.9)	1,834 (40.6)
Cancer therapeutic facility status	Yes	8,494 (80.9)	4,676 (83.6)	609 (77.4)	867 (82.9)	57 (81.4)	3,143 (85.2)	295 (75.1)	3,523 (78.0)
	No	2,009 (19.1)	918 (16.4)	178 (22.6)	179 (17.1)	13 (18.6)	548 (14.8)	98 (24.9)	993 (22.0)
Follow-up period, weeks	Median	120.00	138.29	153.71	147.07	170.79	133.29	123.14	104.36
	Min	4.3	4.4	5.0	4.4	21.9	4.4	4.3	4.3
	Max	617.9	617.9	613.7	586.9	477.1	617.9	565.3	581.3

Data are *n* (%) unless otherwise stated.

A anthracycline, *M* factor metastasis, *Max* maximum, *Min* minimum, *N* factor lymph node, *T* taxane, *T* factor tumor size
